# Barriers to bystander CPR in deprived communities: Findings from a qualitative study

**DOI:** 10.1371/journal.pone.0233675

**Published:** 2020-06-10

**Authors:** Fiona Dobbie, Isa Uny, Douglas Eadie, Edward Duncan, Martine Stead, Linda Bauld, Kathryn Angus, Liz Hassled, Lisa MacInnes, Gareth Clegg

**Affiliations:** 1 Usher Institute, College of Medicine and Veterinary Medicine, University of Edinburgh, Edinburgh, United Kingdom; 2 Institute for Social Marketing and Health, School of Health Sciences, University of Stirling, Stirling, United Kingdom; 3 Nursing Midwifery and Allied Health Professionals Research Unit, Faculty of Health Sciences and Sport, University of Stirling, Stirling, United Kingdom; 4 Resuscitation Research Group, The University of Edinburgh, Edinburgh, United Kingdom; Azienda Ospedaliero Universitaria Careggi, ITALY

## Abstract

**Study aim:**

Rates of out of hospital cardiac arrest are higher in deprived communities. Bystander Cardiopulmonary Resuscitation (BCPR) can double the chance of survival but occurs less often in these communities in comparison to more affluent communities. People living in deprived communities are, therefore, doubly disadvantaged and there is limited evidence to explain why BCPR rates are lower. The aim of this paper is to examine the barriers to administering BCPR in deprived communities.

**Method:**

Mixed method qualitative study with ten single sex focus groups (n = 61) conducted in deprived communities across central Scotland and 18 semi-structured interviews with stakeholders from the UK, Europe and the USA.

**Results:**

Two key themes related to confidence and environmental factors were identified to summarise the perceived barriers to administering BCPR in deprived communities. Barriers related to confidence included: self-efficacy; knowledge and awareness of how, and when, to administer CPR; accessing CPR training; having previous experience of administering BCPR; who required CPR; and whether the bystander was physically fit to give CPR. Environmental barriers focused on the safety of the physical environment in which people lived, and fear of reprisal from gangs or the police.

**Conclusions:**

Barriers to administering BCPR identified in the general population are relevant to people living in deprived communities but are exacerbated by a range of contextual, individual and environmental factors. A one-size-fits-all approach is not sufficient to promote ‘CPR readiness’ in deprived communities. Future approaches to working with disadvantaged communities should be tailored to the local community.

## Introduction

The link between socio-economic deprivation and out of hospital cardiac arrest (OHCA) is widely documented, with the rate of OHCA occurrence being significantly higher in economically deprived areas [1–3]. Reasons for this centre on the risk factors for cardiac arrest being more prevalent in socio-economic deprived populations. These may include: poverty, poor diet (potentially leading to obesity and diabetes), higher incidence of smoking and higher rates of mental illness. [4–7]

Bystander Cardiopulmonary Resuscitation (BCPR) can double the chance of survival from OHCA [8], yet it is performed less often in deprived communities. Findings from Scotland indicate that people living in deprived areas are both more likely to have a cardiac arrest and more likely to have a cardiac arrest earlier in comparison to individuals living in less deprived areas. [9] Similar findings were reported in the USA by Sasson et al (2012) [10] and in England by Becker et al (2019) [11] who identified deprived neighbourhoods as having higher incidences of OHCA with less BCPR. Deprived communities are therefore doubly disadvantaged as they are more susceptible to cardiac arrest and less likely to receive lifesaving BCPR.

Much is known about the barriers to administering BCPR which include: fear of causing harm; lack of CPR skills; and the presentation of the cardiac arrest victim [12–16]. However, existing research has focused on the general population and lacks detail regarding the deprived communities where BCPR is less likely to be performed. For example, a recent systematic review by Chen et al, (2019) [17] of interventions to improve the quality of BCPR made no comment on interventions that would be more appropriate for deprived communities. Becker et al (2019) [11], suggest that people living in more affluent areas are more likely to be CPR trained and, therefore, more confident to administer CPR. Studies have also shown that people with lower education, lower income and lower social grade are less likely to be trained in CPR and feel less confident to perform CPR [18, 19]. For example, Dobbie et al (2018) found that people with a higher social grade were more likely to be trained in CPR than those with a lower social grade (57% compared with 48%). Similar findings were also found when they looked at confidence to administer CPR if talked through a by a call handler; respondents with higher social grade were more confident than those with a lower social grade (86% compared with 78%) [18].

A recent systematic review by Uny et el, 2019 [20] sought to examine the barriers to administering BCPR in deprived communities. Nineteen studies were included in the review, but just three (all from the USA) considered deprived communities. The remaining 16 included some deprivation measures but were classed as low-relevance due the lack of segmentation by deprivation. This means that findings were not specifically analysed by socio-economic status, resulting in a limited understanding of whether there are specific factors that are more relevant to deprived communities or not. This paper presents findings from a qualitative study to explore the barriers to administering BCPR in deprived communities. In doing so it will inform future initiatives to improve the rate of BCPR in deprived communities, not just in the UK, but also in Europe and North America.

## Method

Qualitative research was conducted as part of a larger mixed-method study to design an evidence based intervention to improve the rate of BCPR in deprived communities in Scotland [20]. Ten single sex focus groups were conducted in SMID (Scottish Index of Multiple Deprivation) areas 1 and 2 which represent the most deprived parts of Central Scotland [21]. Groups were single sex because administering CPR to women has been found to be a potential barrier pertinent to men [22]. Participants were recruited via local community groups and face-to-face street recruitment. To ensure diversity recruitment quotas were set and are summarised in Table 1. Across the sample overall at least six participants were from black and minority ethics groups and at least 15 had CPR training and a further 15 had no CPR training.

**Table 1:** Focus group sample.

Group ID	Social grade*	Gender	Age	Area	Number of participants
**01**	C2DE	Female	18–24	Glasgow	6
**02**	C2DE	Female	25–44	Edinburgh	6
**03**	C2DE	Female	45–59	Glasgow	8
**04**	C2DE	Female	60 and over	Edinburgh	5
**05**	C2DE	Male	18–24	Edinburgh	5
**06**	C2DE	Male	25–44	Glasgow	8
**07**	C2DE	Male	45–59	Edinburgh	5
**08**	C2DE	Male	60 and over	Glasgow	5
09*	C2DE	Mixed	NA	Glasgow	6
10*	C2DE	Mixed	NA	Glasgow	7
**TOTAL**					61

* Social grade was determined using the National Readership Survey (NRS)

** Participants were actively engaged in their community (for example, through tenants’ associations or other community groups, community ‘activators’)

To augment findings from local communities and offer a broader perspective of the potential barriers that focus groups participants may not have perceived or articulated, semi-structured interviews (n = 18) were conducted with a range of stakeholders who had experience in BCPR or working with deprived communities. Stakeholders were selected through discussion with the advisory group and included: academics (n = 6); medicine and emergency service workers (n = 4); public and third sector (i.e. non-governmental and non-profit-making organizations or associations, including charities, voluntary and community groups) workers (n = 8).

The study was approved by the General University Ethics Panel at Stirling University (reference: GUEP297) and written informed consent was obtained from all participants. Interviews and focus groups were audio-recorded and transcribed verbatim, with observation notes written after each group. A structured, thematic analysis based on systematic coding of verbatim transcripts organised and managed via QSR Nvivo12 was conducted [23]. Coding frames were jointly developed, piloted and amended by two members of the research team FD and IU. Coding was conducted by FD, IU, DE and KA Findings are reported using the Standards for Reporting Qualitative Research (SRQR) guidelines [24].

## Results

Focus group and stakeholder findings were coded into six themes to summarise the perceived barriers to administering BCPR in deprived communities: self-efficacy; knowledge and awareness; training; experience; who required CPR; and bystander health. Of particular importance was the overarching barrier of confidence which weaved its way through each of these themes.

*" Confidence definitely and a lack of self-worth…*.. *People in this area very much see themselves as*, *‘oh well that’s no[t] me*, *I’m no[t] capable of doing that*, *I don’t have the skills to do that*.*’                        (Stakeholder public and third sector 08)*

This lack of confidence was perceived to be compounded in deprived communities by other daily struggles such as: unemployment, debt, poor housing, mental ill health, poor physical health, addiction, and domestic abuse.

*" I can tell you for a fact the reason people aren’t learning CPR is because they are worrying about staying alive… Worrying about CPR training is only one of 100 other things they have to worry about on a daily basis*.

                                    *(Stakeholder academic 16*)*​*

### Barriers related to a lack of confidence

The perception of **self-efficacy** in regards to the skills needed to perform BCPR was lacking in deprived communities, with a common misperception that CPR should be administered by someone more skilled (e.g. a doctors or a nurse).

*" I think sometimes in this area*, *people in this area*, *very much think that because they are from [a deprived area] they will never achieve anything…*. *CPR to people in this area is very much something that a doctor does in the hospital or a nurse*.*”                (Stakeholder public and third sector 08)*

**Knowledge** and **awareness** of when and how to administer CPR was a key barrier to bystander confidence. Stakeholders commented that the effect of this barrier was more pronounced in deprived communities because people tended to have less educational attainment and fewer opportunities to take part in CPR training. Linked to this was a lack of confidence from bystanders that they would recognise when CPR was required.

*Interviewer*: *“Do you think you would have known if she needed CPR or not?”*

*Participant*: *“I wouldnae [would not] have known*, *that’s why I would have called for the professionals*.*”                                    (Focus group 2*, *female 25–44)*

Stakeholders commented that this lack of knowledge was evident in the inappropriate use of the terms ‘cardiac arrest’ and ‘heart attack’, with bystanders not knowing that they have different outcomes. This finding was confirmed by the focus group discussions where it was common for participants to assume that a heart attack and cardiac arrest were the same thing.

*Interviewer*: *“So do people think there’s a difference between a heart attack and a cardiac arrest?*

*Participant*: *It’s the same think*, *I think*. *My da’s [dad has] took three and my da’s [dad’s] brother took one and my uncle*, *he’s in hospital the noo [now]*.*”                                        (Focus group 7*, *male 45–59)*

Stakeholders also commented that the term CPR was confusing, suggestive of a medical intervention rather than a simple first-aid manoeuvre. This confusion was also evident in focus group discussion including those who had been CPR trained.

*"I don’t know what it is now [how to administer CPR]*, *it’s changed a couple of times over the course of 10 years or so*.*”                            (Focus group 9*, *mixed*, *community)*

Linked to this, was a lack of awareness around what to expect when administering BCPR. Unsurprisingly, participants who had no CPR training or experience of witnessing CPR did not know what to expect (e.g. cracking ribs or physical presentation of the victim: colour, noise, potential for vomit) which was perceived to be ‘off-putting’.

One potential route to address the gaps in knowledge and awareness discussed above is to offer BCPR **training** and refreshers for people who had been trained previously. However, analysis highlighted that training delivery in deprived communities would require careful consideration. For example, stakeholders expressed a view that bystanders may not feel adequately skilled or equipped to administer BCPR unless they had received formal training–i.e. by a professional rather than watching something on television or online.

*"Sometimes there is a barrier for them to get formal training*. *Formal training isn’t a necessity of any kind*, *but I would think that perhaps they feel that because they haven’t had formal training*, *they feel as though they can’t do it*.*"*

                            (*Stakeholder public and third sector 03*)

**Past experience** administering BCPR was viewed as an important factor affecting confidence especially if the victim did not survive. This was evident in the lived experience of one focus group participant who found a situation requiring BCPR so traumatising that they later resigned from their post as a first aider in work.

*"I was a first aider for about 10 year*…*But my neighbour hung himself and I had to gie [give] him CPR…*.*we cut him doon [down]*, *we put him doon [down] and he still died…*.*we tried our best…*. *I went into work and handed my first aid bag in*. *I couldnae [could not] do it [anymore]”*.

                                *(Focus group 7*, *male 45–59)*

Stakeholders also recognised that past CPR experience could affect future confidence, acknowledging a need for more follow-up support to be offered to bystanders who attempt BCPR.

*"If somebody’s had a bad experience [administering CPR]*, *their confidence is completely shot in regards that there’s no way they would want to administer CPR again"*

                                    (*Stakeholder public and third sector 05*)

Focus group discussion suggested that **who required CPR** was another important consideration. On the one hand, there was a view that bystanders would be more inclined to administer CPR to someone they knew, a relative for instance. However, there was also comment that it could be harder because they may be more likely to panic,

*"I actually think it’s easier–in a situation that is lifesaving*, *it’s probably easier to stay calm if you don’t know the person*, *than if you are emotionally involved with the person*. *I wouldn’t imagine myself being good and proactive in giving to someone I am very close to”*.

                              *(Focus group 10 mixed*, *community)*

Due to physical demands of CPR, it is not surprising that focus group participants and stakeholders recognised that the **health of the bystander** (especially if it was poor) could affect their confidence to administer CPR. This barrier was perceived to be more acute in deprived areas where people have more chronic health conditions such as COPD,

*"I think people don’t want or can’t use CPR*, *do you know if somebody has maybe got a disability*, *or if they’re frail*, *because we’re all fairly like fat and overweight”*.    *(Focus group 1*, *female*, *18–24)*

### Environmental barriers

Results presented, so far, have focused on the factors that affect confidence to administer BCPR. Whilst these factors are also likely to be relevant to other populations, for deprived communities (who are situated in the context of poverty) this makes them more pervasive and challenging to address. However, our analysis also identified environmental factors that were perceived to be specific barriers to administering BCPR in deprived communities. First, was the perception that bystanders may be less willing to help someone who they believed to be a drug addict or alcoholic, a population group that were considered to be more common in deprived communities

*"There’s a lot of people who end up lying in back courts*, *and in streets and you would think that’s drugs and walk by them*. *Know what I mean”*.      *(Focus group 4*, *female*, *60+)*

This was in contrast to someone’s gender, race or sexuality which were not be seen as barriers to administering CPR.

*"If they [bystander] were in cardiac arrest*, *regardless of their religion*, *their race*, *or sex*, *their political beliefs*, *I would do CPR on anybody and*..*…I don’t think for one second somebody would not do CPR on somebody because they were wearing a burka*, *or because they were gay*.*”                                (Focus group 5*, *male*, *60+)*

Second, was a fear for their own personal safety if they decided to help in what was seen as a high-risk situation,

*"The other night*, *I was walking down [the road] at half past two in the morning*, *and there was someone lying unconscious in the street and I went over to help thinking he will be in some kind of…until someone went and kicked him in the head*. *I’m like okay second thoughts just walk away”*.                                *(Focus group 5*, *male*, *18–24)*

A similar view came from a stakeholder who commented that bystanders living in deprived areas may be fearful of performing BCPR because of the potential repercussions, especially if the victim was part of a gang or a well-known family with criminal connections,

*"In this area*, *there is a lot of violence*, *there is a drug culture*, *and gang culture and it’s very much a case of ‘oh I’m no[t] going over there*, *you don’t know who he’s related to*, *or the person standing next to him might have a knife*, *or what if I break his rib and then I end up getting comeback from it*.*"            (Stakeholder public and third sector 08)*

In addition to fear for their own personal safety, there was also a fear of the police. This was discussed from two perspectives: 1) if the bystander had a criminal record they may not want to attempt CPR in case it brought them into contact with the police; 2) if the victim and the bystander were implicated as a drug user or a related crime, the bystander may decide to walk away for fear of being blamed for the victim requiring CPR and ultimately his or her death.

*"I was just thinking aboot [about] people in addiction right…I can tell you many stories aboot [about] the person who is still able-bodied but the fear kicks in and they just run because the fear of being blamed for that person’s death potentially*.*”*

                              *(Focus group 6*, *male*, *25–44)*

## Discussion

Previous studies examining barriers to administering bystander CPR have taken a general population perspective, with limited discussion of whether these barriers are relevant to people living in deprived communities or not. As a result, existing initiatives to improve the rate of bystander CPR may be failing to consider the context in which bystanders live. Recognising the context in which an initiative is situated creates a greater understanding of how, and why it is successful (or unsuccessful) and aids assessment of transferability to different settings or populations [25, 26]. Without this understanding it is difficult to gauge whether existing initiatives (such as mass media campaigns) to improve the rate of BCPR are effective in the communities that need them most.

Numerous studies examining why members of the general public may not feel comfortable administering BCPR highlight confidence as one of several explanatory factors [1, 11–14, 16, 18].

However, our analysis highlighted that the overarching barrier to administering CPR in deprived communities was confidence, which impacted a bystander’s self-belief that they could and should attempt BCPR. Whilst it can be argued that confidence underpins barriers to administering BCPR in both the general population and the deprived population, it is exacerbated in deprived communities because of the other daily struggles people may face which impact confidence and self-efficacy more generally [27].

This study also identified barriers that were perceived to resonant more with people living in deprived communities. These related to the physical environment in which people lived (i.e. not feeling safe to offer help) and fear of reprisal from gangs or the police. These findings concur with work by Sasson (2013) who also identified environmental factors relating to personal safety (e.g. higher perceptions of risk due to violence or crime) as specific barriers to administering BCPR in deprived communities [28].

Tackling these barriers is key if local people living in deprived communities are to become ‘CPR ready’. One way to achieve this is to promote awareness of Good Samaritan Laws’ which are in place across Europe, America, Canada and Australia. Despite variation by country, these laws protect the bystander from prosecution due to unintentional injury or death and can offer protection from criminal prosecution if the bystander was in possessions of illegal drugs or associated paraphernalia [29].

A key priority, not just for Scotland but worldwide, is to improve the rate of BCPR [30–33]. Findings from this study have highlighted two further recommendations to help achieve this in deprived communities. First, we argue that a one-size-fits-all approach is not sufficient to improve the rate of BCPR in deprived communities. Rather, more tailored initiatives are required to promote ‘CPR readiness’. We propose three components to being CPR ready: 1) having the **belief** that bystanders should attempt CPR; 2) feeling **confident** and safe to give CPR and; 3) having the **practical skills** to administer CPR. Second, any initiative to promote CPR readiness needs to take cognisance of building confidence to enable bystanders to proactively respond to an emergency situation requiring CPR. This will require careful thought when viewed from the lens of the additional challenges people living in deprived communities face. For example, as noted by Sasson, offering traditional CPR training classes will only reach local people who can afford to attend (e.g. travel and child care costs) and have the time and motivation to attend [28, 34]. Other more informal and innovative approaches using social marketing and social networks, for example, may be more effective. Further research is underway by the authors to explore how these initiatives could be delivered, with existing guidelines emphasising the importance of being theoretically informed and developed with the community [35].

A strength of this study is its use of qualitative methods which adds greater depth of understanding to an under researched area. However a limitation is that findings were confined to deprived communities in two cities in Scotland, which means finding are not inclusive of rural areas. Further, as noted in other papers on similar topics [11] our findings are mostly based on the perceived barriers to BCPR from people who did not have any actual experience of being in a situation requiring BCPR. Nonetheless, our sample was carefully chosen to include a range of people who had different levels of CPR experience and also included professionals who either worked in deprived communities, responded to OHCA or offered CPR training which ensured a range of perspectives.

## Conclusion

Barriers to administering Bystander CPR identified in the general population are relevant to people living in deprived communities but are exacerbated by a range of contextual, individual and environmental factors. A one-size-fits-all approach is not sufficient to improve the rate of CPRBCPR in deprived communities. Future approaches to working with disadvantaged communities should be tailored and seek to address the barriers identified in this paper.

## Supporting information

S1 File Focus group topic guide. (DOCX)

S2 File Stakeholder topic guide. (DOC)

## References

[1] Vaillancourt C, Lui A, De Maio VJ, Wells GA, Stiell IG. Socioeconomic status influences bystander CPR and survival rates for out-of-hospital cardiac arrest victims. Resuscitation. 2008;79(3):417–23. 10.1016/j.resuscitation.2008.07.012 18951678

[2] Mitchell M, Stubbs BA, Eisenberg MS. Socioeconomic Status Is Associated with Provision of Bystander Cardiopulmonary Resuscitation. Prehosp Emerg Care. 2009;13(4):478–86. 10.1080/10903120903144833 19731160 PMC3041986

[3] Sasson C, McNally B, Hinchey P, Gonzales L, Persse D, Root E. A Tale of Two Cities: The Role of Neighborhood Socioeconomic Status in Spatial Clustering of Bystander CPR in Austin and Houston. Circulation. 2011;124(21). 10.1016/j.resuscitation.2013.01.007 PMC3762246 23318916

[4] Masterson S, Teljeur C, Cullinan J, Murphy AW, Deasy C, Vellinga A. Out-of-hospital cardiac arrest in the home: can area characteristics identify at-risk communities in the republic of ireland? 2018;17(1). 10.1186/s12942-018-0126-z 29458377 PMC5819205

[5] Reinier K, Thomas E, Andrusiek DL, Aufderheide TP, Brooks SC, Callaway CW, et al Socioeconomic status and incidence of sudden cardiac arrest. CMAJ: Canadian Medical Association journal = journal de l'Association medicale canadienne. 2011;183(15):1705 10.1503/cmaj.101512 21911550 PMC3193117

[6] Pujades-Rodriguez M, Timmis A, Stogiannis D, Rapsomaniki E, Denaxas S, Shah A, et al Socioeconomic Deprivation and the Incidence of 12 Cardiovascular Diseases in 1.9 Million Women and Men: Implications for Risk Prediction and Prevention.(Research Article). PLoS ONE. 2014;9(8). 10.1371/journal.pone.0104671 PMC4140710 25144739

[7] Berdowski J, Berg RA, Tijssen JGP, Koster RW. Global incidences of out-of-hospital cardiac arrest and survival rates: Systematic review of 67 prospective studies. Resuscitation. 2010;81(11):1479–87. 10.1016/j.resuscitation.2010.08.006 20828914

[8] Riva G, Ringh M, Jonsson M, Svensson L, Herlitz J, Claesson A, et al Survival in Out-of-Hospital Cardiac Arrest After Standard Cardiopulmonary Resuscitation or Chest Compressions Only Before Arrival of Emergency Medical Services: Nationwide Study During Three Guideline Periods. Circulation. 2019;139(23):2600–9. 10.1161/CIRCULATIONAHA.118.038179 30929457

[9] Scottish Government The Scottish G. Initial Results of the Scottish Out-of-Hospital Cardiac Arrest Data Linkage Project. APS Group; 2018.

[10] Sasson C, Cudnik MT, Nassel A, Semple H, Magid DJ, Sayre M, et al Identifying High‐risk Geographic Areas for Cardiac Arrest Using Three Methods for Cluster Analysis. Academic Emergency Medicine. 2012;19(2):139–46. 10.1111/j.1553-2712.2011.01284.x 22320364

[11] Becker TK, Gul SS, Cohen SA, Maciel CB, Baron-Lee J, Murphy TW, et al Public perception towards bystander cardiopulmonary resuscitation. Emergency Medicine Journal. 2019. 10.1136/emermed-2018-208234 31473603

[12] Aaberg AM, Larsen CE, Rasmussen BS, Hansen CM, Larsen JM. Basic life support knowledge, self-reported skills and fears in Danish high school students and effect of a single 45-min training session run by junior doctors; a prospective cohort study. Acta Veterinaria Scandinavica. 2014;22(1):24. 10.1186/1757-7241-22-24 PMC4022325 24731392

[13] Riegel B, Mosesso VN, Birnbaum A, Bosken L, Evans LM, Feeny D, et al Stress reactions and perceived difficulties of lay responders to a medical emergency. Resuscitation. 2006;70(1):98–106. 10.1016/j.resuscitation.2005.10.029 16753251

[14] Kanstad BK, Nilsen SA, Fredriksen K. CPR knowledge and attitude to performing bystander CPR among secondary school students in Norway. Resuscitation. 2011;82(8):1053–9. 10.1016/j.resuscitation.2011.03.033 21531067

[15] Sasson AMC, Rogers LM, Dahl LJ, Kellermann LA. Predictors of Survival From Out-of-Hospital Cardiac Arrest: A Systematic Review and Meta-Analysis. Circulation: Cardiovascular Quality and Outcomes. 2010;3(1):63–81. 10.1161/CIRCOUTCOMES.109.889576 20123673

[16] Vaillancourt C, Stiell IG, Wells GA. Understanding and improving low bystander CPR rates: A systematic review of the literature. Canadian Journal of Emergency Medicine. 2008;10(1):51–65. 10.1017/s1481803500010010 18226319

[17] Chen K-Y, Ko Y-C, Hsieh M-J, Chiang W-C, Ma MH-M. Interventions to improve the quality of bystander cardiopulmonary resuscitation: A systematic review.(Research Article)(Report). PLoS ONE. 2019;14(2):e0211792 10.1371/journal.pone.0211792 30759140 PMC6373936

[18] Dobbie F, MacIntosh AM, Clegg G, Stirzaker R, Bauld L. Attitudes towards bystander cardiopulmonary resuscitation: Results from a cross-sectional general population survey. PLoS ONE. 2018;13(3):e0193391 10.1371/journal.pone.0193391 29513722 PMC5841784

[19] Blewer AL, Ibrahim SA, Leary M, Dutwin D, McNally B, Anderson ML, et al Cardiopulmonary Resuscitation Training Disparities in the United States. Journal of the American Heart Association: Cardiovascular and Cerebrovascular Disease. 2017;6(5). 10.1161/JAHA.117.006124 PMC5524119 28515114

[20] Uny I, Duncan E, Stead M, Critchlow N, Eadie D, MacInnes L, er al Let's be CPR Ready: a development study 2019 [Available from: https://www.cso.scot.nhs.uk/wp-content/uploads/Hips1710.pdf.

[21] https://www.isdscotland.org/Products-and-Services/GPD-Support/Deprivation/SIMD/.

[22] Kramer CE, Wilkins MS, Davies JM, Caird JK, Hallihan GM. Does the sex of a simulated patient affect CPR? Resuscitation. 2015;86:82–7. 10.1016/j.resuscitation.2014.10.016 25447437

[23] Ritchie J, Lewis J, McNaughton Nicholls C, Ormston R. Qualitative research practice: a guide for social science students and researchers. Second edition / edited by Jane Ritchie, Lewis Jane, Carol McNaughton Nicholls, Rachel Ormston. ed. Los Angeles: Los Angeles: SAGE; 2014.

[24] O’brien CB, Harris BI, Beckman JT, Reed AD, Cook AD. Standards for Reporting Qualitative Research: A Synthesis of Recommendations. Academic Medicine. 2014;89(9):1245–51. 10.1097/ACM.0000000000000388 24979285

[25] Craig P. Taking account of context in population health intervention research: guidance for producers, users and funders of research. Di Ruggiero E, Frohlich KL, Mykhalovskiy E, White M, editors.

[26] Moore G, Audrey S, Barker M, Bond L, Bonell C, Cooper C, et al Process evaluation in complex public health intervention studies: the need for guidance. Journal of Epidemiology and Community Health. 2014;68(2):101 10.1136/jech-2013-202869 24022816 PMC3892708

[27] Brady D, Burton L. The Oxford handbook of the social science of poverty. First edition ed. New York, NY: New York, NY: Oxford University Press; 2016.

[28] Sasson SC, Haukoos HJ, Bond HC, Rabe HM, Colbert HS, King HR, et al Barriers and Facilitators to Learning and Performing Cardiopulmonary Resuscitation in Neighborhoods With Low Bystander Cardiopulmonary Resuscitation Prevalence and High Rates of Cardiac Arrest in Columbus, OH. Circulation: Cardiovascular Quality and Outcomes. 2013;6(5):550–8. 24021699 10.1161/CIRCOUTCOMES.111.000097 PMC3886185

[29] Jakubowski A, Kunins HV, Huxley-Reicher Z, Siegler A. Knowledge of the 911 Good Samaritan Law and 911-calling behavior of overdose witnesses. Substance Abuse. 2018;39(2):233–8. 10.1080/08897077.2017.1387213 28972445

[30] Mathiesen WT, Bjarshol CA, Kvalay JT, Sareide E. Effects of modifiable prehospital factors on survival after out-of-hospital cardiac arrest in rural versus urban areas.(Report). Critical Care. 2018;22(1). 10.1186/s13054-018-1946-8 29669574 PMC5907488

[31] Global Resuscitation Alliance. Improving Survival from Out-of-Hospital Cardiac Arrest: A Call to Establish a Global Resuscitation Alliance. 2016.

[32] Nadarajan GD, Tiah L, Ho AFW, Azazh A, Castren MK, Chong SL, et al Global resuscitation alliance utstein recommendations for developing emergency care systems to improve cardiac arrest survival. Resuscitation. 2018;132:85–9. 10.1016/j.resuscitation.2018.08.022 30171975

[33] Scottish Government The Scottish G. Out-Of-Hospital Cardiac Arrest—A Strategy For Scotland. APS Group; 2015.

[34] Sasson C, Haukoos JS, Ben-Youssef L, Ramirez L, Bull S, Eigel B, et al Barriers to Calling 911 and Learning and Performing Cardiopulmonary Resuscitation for Residents of Primarily Latino, High-Risk Neighborhoods in Denver, Colorado. Annals of Emergency Medicine. 2015;65(5):545–52.e2. 10.1016/j.annemergmed.2014.10.028 25481112 PMC4866505

[35] Craig P, Dieppe P, Macintyre S, Michie S, Nazareth I, Petticrew M. Developing and evaluating complex interventions: The new Medical Research Council guidance. International Journal of Nursing Studies. 2013;50(5):587 10.1016/j.ijnurstu.2012.09.010 23159157

