# Correction: Barriers to bystander CPR in deprived communities: Findings from a qualitative study

**DOI:** 10.1371/journal.pone.0244104

**Published:** 2020-12-10

**Authors:** Fiona Dobbie, Isa Uny, Douglas Eadie, Edward Duncan, Martine Stead, Linda Bauld, Kathryn Angus, Liz Hasseld, Lisa MacInnes, Gareth Clegg

The eighth author's name is spelled incorrectly. The correct name is: Liz Hasseld
