# Supplementary material for: Barriers to bystander CPR in deprived communities: Findings from a qualitative study
Source: PLoS One. 2020 Jun 10;15(6):e0233675. doi: 10.1371/journal.pone.0233675 (PMC7286503; doi:10.1371/journal.pone.0233675)
Supplement: S1 File — (DOCX) [file pone.0233675.s001.docx]

**Bystander CPR topic guide for focus group discussion**

***Aims of the focus group (FGD) discussion:***

- Understanding the specific barriers and facilitators to administering bystander CPR in deprived communities
- Understanding what it means to be CPR ready
- Better understanding of the role and potential contribution of social networks to improve the rate of bystander CPR in deprived communities.

**INTRODUCTION/ WELCOME**

- **Thank people for coming ( we know you are busy; your views are important to us)**
- **Format of the discussion (respect each other; one talking at a time; everyone has a chance to speak: what is said in discussion is confidential)**
- **Discussion is recorded and we will take notes***.* **Explain *about* reporting, use of quotations, anonymization.**
- **No right or wrong answers ( everyone’s views matter; you can disagree with others)**
- **Withdrawal at any time from interview as whole, or in not answering particular questions ( particularly if hard for you; you can also take a break and come back)**
- **Timing of group discussion interview (around 60 minutes)**
- **Check if participants have any questions? Happy to proceed?**
- **Obtain consent, leave a copy of the consent form with the participant and return the other signed copy to the office.**
- **Get participants to fill in the demographics questionnaire**
- **Also flag that we are looking for one lay person to join the advisory group**

**KEY AREAS FOR EXPLORATION IN FOCUS GROUPS**

1. **Introductions**

Go round the group ask them to introduce themselves and tell us about a typical day during the week (for those who work probe for detail on their job title)

1. **Awareness, attitudes and perceptions of CPR**

- What does CPR mean to you?

[let the group give spontaneous answers first and then clarify the definition] - *CPR stands for cardiopulmonary resuscitation. It is given to someone who is in cardiac arrest (which means they are unconscious and have stopped breathing). It helps to pump blood around the person's body when their heart can’t and is vital to saving their life.)*

- How would you know if someone needs CPR?
  - *How easy is it to determine?*
  - *What are the signs and symptoms?*
- Does anyone know how do it?
  - *how do you do it?*
  - *Is chest compressions / pressing on the chest enough or do you need to do mouth to mouth (rescue breaths) as well*
- Has anyone seen or read about CPR (this could be in real life or on a TV programme or movies?
- Who can give CPR?
  - *anyone or specific types of people like paramedics, emergency services, NHS staff etc.*
  - *Do they need to be trained in CPR? If yes, what would this training look like? [i.e. what type of training do they need - attend a training course, learned about it from a leaflet, online line, TV?]*

1. **Barriers and facilitators** (Here we want to explore what people would do would if someone collapsed in front of them with a cardiac arrest).
2. **First show a short video of someone having a cardiac arrest (note only show up until the person collapses from a cardiac arrest).**

You can choose from either the Lifesaver video app (<https://life-saver.org.uk>). (we have selected the following 3 clips that would be suitable for the different age groups).

- Jake (collapses in the subway, he is with a couple friends)- **may work well with 30+ age group**
- Harry (collapses at home, playing football with a few friends)- **may work well with 16-25 age group male**

**OR** Use the scenario from the video: <https://vimeo.com/172116741> . Show only the first 1minute 14s minutes-  **may work better with 45-60 + age groups**

- - Middle- age man goes to work and collapses on the street with no one around.

OR **a short written scenario (NOTE only use this if you can’t play a video, no internet access for example)**

- *Imagine that you are walking down the street to the shops and you see a person collapse. You come to look at them and they seem unconscious, not breathing and have no pulse. You are only person around. How do you think you would feel at this point?*
- If this was a real life situation some people would help and some people would not, what do you think the reasons for this are?

**b- Secondly, follow up with questions** about the people/ situation in the video chosen for the group demographics, to open up the discussion, help people project:

- *Start by asking for their initial thoughts/reactions – you are trying to tease out how they would respond and why.*
- *After this initial discussion follow-up with:*
  - *How do you think you would feel at this point?*
  - If this was a real life situation some people would help and some people would not, what do you think the reasons for this are?
- *Is there a specific type of person that people may be less comfortable to help? (e.g. a homeless person, someone who was drunk, a drug addict, someone from a different race /culture?)*
- *For those who would help what would they actually do? (i.e. in practical terms dial 999, hand only CPR)? Why is this?*
- *For those who would not do any more than dial 999, why is this?*
- *Is there more you would like to be able to do? If yes, what? How could this happen?*

1. **Being CPR ready**

Explain that the point of this research is to save lives by improving the survival rate from a cardiac arrest in Scotland. To do this we want to make more people ‘CPR ready.’ This means we want people to be willing and confident to have a go at CPR.

- - *How do we do this?*
  - *What would work/not work in your community?*
  - *If training not mentioned spontaneously probe specifically on this – e.g.;*
    - *What does training mean (i.e. what does it look like/involve - attending a course or watching a video on you tube?)*
    - *Does it matter what type of training people have had?*
    - *What training have they had (i.e. when, and why, how was it delivered, how long did it take?)*
- What would it take for you to have a go if you needed to?

1. **Close**

- Any further comments
- Follow-up for workshops
