# Supplementary material for: Barriers to bystander CPR in deprived communities: Findings from a qualitative study
Source: PLoS One. 2020 Jun 10;15(6):e0233675. doi: 10.1371/journal.pone.0233675 (PMC7286503; doi:10.1371/journal.pone.0233675)
Supplement: S2 File — (DOC) [file pone.0233675.s002.doc]

**Bystander CPR topic guide for stakeholder interview**

## Aims of the interview:

- Better understanding of existing work to improve the rate of bystander CPR and it’s applicability to deprived communities
- Understanding the specific barriers and facilitators to administering bystander CPR in deprived communities
- Better understanding of how to measure and improve the rate of bystander CPR in deprived communities
- Better understanding of the role and potential contribution of social networks to improve the rate of bystander CPR in deprived communities.

**Introduction/Recap**

**Background Information**

Recap purpose of study

**Interview Format**

Format of interview (open questions, hearing their views)

No right or wrong answers – their views are important

Confidentiality and limits around disclosures of harm

Withdrawal at any time from interview as whole, or in not answering particular questions

Timing of interview (around 45-60 minutes)

**Recording of Interview**

Digital recording of interviews – check they are happy with this

Report, use of quotations, anonymisation

Check if participant has any questions?

Check if happy to proceed?

**Consent**

Obtain consent, leave a copy of the consent form with the participant and return the other signed copy to the office.

**1: Current role and experience**

- Please tell me about your current role?
  - Probe: how long in post?
- Does your current or previous role have any involvement with bystander CPR (e.g. delivering training, develop strategies/policy/knowledges/ideas to improve the rate of bystander CRP?
  - Probe: a) in general, b) deprived communities

**2: Terminology and measuring bystander CPR**

- The purpose of this research study is to design an intervention to improve the rate of bystander CPR in deprived communities. **What does the term ‘*improve the rate of bystander CPR’* mean to you**? [we are trying to tap into the complexity of designing an intervention to improve the rate of bystander CPR – e.g. delivering training alone may not be effective if people do not feel confident and have been trained some time ago]
- Will increasing the uptake of CPR training improve the rate of bystander CPR?
  - Probe reason for answer
- What does the term ‘CPR ready’ mean to you? [rather than simply have someone CPR trained, CPR ready means having the skills (i.e. CRP training) but also the belief and confidence that it is the right thing to do even if they were trained some time ago)
- What would be being CPR ready look like?
- What would have to happen to make someone CPR ready?
- What type of training/advertising campaign/initiative/intervention would be required to encourage more people to become CPR ready?
- How can we measure the rate of bystander CPR in Scotland?
  - Probe if they know of monitoring systems already in place

**3: Barriers and facilitator to administering bystander CPR in deprived communities**

- What do you think are the main reasons someone would attempt bystander CPR?
- What do you think are the main reasons someone would not attempt bystander CPR?
- Are these reasons the same for people living in deprived communities or not?
  - Probe reason for answer (i.e. are there specific barriers and facilitators for people living in deprived communities)

**4: Existing work to improve bystander CPR**

- What work are you aware of that has tried to either promote, raise awareness /confidence or change attitudes towards bystander CPR?
- Probe:
- What was it?
- When was this work conducted? – is it still ongoing?
- Who was the target group? E.g. any specific age, gender, ethnicity, employment status, socio-economic group?
- Are you aware of anything specifically tailored for deprived communities? If yes, expand.
- Thinking about the existing work you are aware of, is any of it transferable to deprived communities? Probe reason for answer.
- Has any of this work been monitored or evaluated? If yes, ask for references.

**5: Improving the rate of bystander CPR in deprived communities**

- How do you think we can improve the rate of bystander CPR in deprived communities?
- Can we improve the rate of bystander CPR in deprived communities by boosting the number of people trained in CPR?
  - Probe reason for answer. If no, why not? What else do we need to do?
- How important are social networks (e.g. friends, family, and colleagues) in shaping attitudes, confidence and willingness to administer bystander CPR in deprived communities?
  - Probe reason for answer, what role do they have? Is it positive or negative?
- What role could social networks have in improving the rate of bystander CPR in deprived communities?
  - Probe for any examples

**6: Any other comments/questions?**

**Thank and close**
